# Risk of cutaneous adverse events in cancer patients treated with phosphatidylinositol‐3‐kinase inhibitors: A systematic review and meta‐analysis of randomized controlled trials

**DOI:** 10.1002/cam4.5153

**Published:** 2022-08-19

**Authors:** Yushu Wang, Zhuo Ma, Zhuoling An, Yi Zhang, Xin Feng, Xiaojia Yu

**Affiliations:** ^1^ Department of Pharmacy Beijing Chao‐Yang Hospital, Capital Medical University Beijing China; ^2^ Department of Pharmacy Beijing Obstetrics and Gynecology Hospital, Capital Medical University. Beijing Maternal and Child Health Care Hospital Beijing China

**Keywords:** cancer, cutaneous adverse events, PI3K inhibitors, randomized controlled trials, rash

## Abstract

**Background:**

Cutaneous adverse effects (AEs) are common following the phosphoinositide‐3‐kinase (PI3K) inhibitors treatment. We aim to estimate the incidence and risk of PI3K inhibitor‐related cutaneous AEs.

**Methods:**

The protocol was submitted to the PROSPERO registry. We searched ClinicalTrials.gov and international databases up to July 29, 2022. Meta‐analysis was conducted by using risk ratios (RRs) with 95% confidence intervals (CIs).

**Results:**

Fourteen randomized controlled trials (RCTs) comprising 3877 patients were analyzed in this study. Compared with control arms, PI3K inhibitors showed a significant increase in the risk of all‐grade rash, high‐grade rash, and serious rash events (RR 2.29, 95% CI 1.58–3.31, *p* < 0.00001; RR 9.34, 95% CI 4.21–20.69, *p* < 0.00001; RR 5.11, 95% CI 2.11–12.36, *p* = 0.0003). The overall incidences of all‐grade rash and high‐grade rash were 26.2% (592/2257) and 4.4% (66/1487). Subgroup analyses of all‐grade rash according to cancer types and PI3K inhibitor assignations identified the significant associations. PI3K inhibitors also significantly increased the risk of pruritus and dry skin (RR 1.63, 95% CI 1.14–2.33, *p* = 0.007; RR 3.34, 95% CI 2.30–4.85, *p* < 0.00001), with incidences of 13.4% (284/2115) and 9.8% (141/1436) in the treatment group.

**Conclusion:**

There is a significantly increased risk of some cutaneous AEs in patients using PI3K inhibitors. Advance intervention is recommended in case of severe and life‐threatening events. Further research is required to investigate the risk factors and pathogenesis.

## Graphical abstract

There is a significant increased risk of some cutaneous AEs in patients using PI3K inhibitors. Further research are required to investigate the risk factors and pathogenesis.

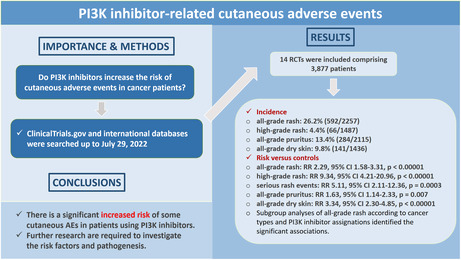

## 1 INTRODUCTION

Phosphoinositide‐3‐kinase (PI3K) signaling is able to regulate cell proliferation, differentiation, migration, and apoptosis. Due to its crucial function across cancers, the PI3K pathway has always been the research emphasis of anticancer drugs. Inhibitors of PI3K not only displayed activity as monotherapy but also obtained efficacy results in combination with other treatments.^1^, ^2^

Idelalisib was the first PI3K inhibitor to be approved by the United States Food and Drug Administration (FDA) for chronic lymphocytic leukemia (CCL), follicular lymphoma (FL), and relapsed small lymphocytic lymphoma (SLL).^3^ Copanlisib was the next approved for relapsed FL, followed by the approval of duvelisib in relapsed or refractory CLL or SLL.^4^, ^5^ In addition to their indications in hematologic cancers, another PI3K inhibitor alpelisib was also approved for the PIK3CA‐mutated advanced breast cancer in 2019.^6^, ^7^ Despite promising results, toxicities of PI3K inhibitors that can lead to potential detriments in overall survival (OS) have been reported^8^, ^9^, ^10^ and have raised many concerns in a recent Oncologic Drugs Advisory Committee (ODAC) Meeting convened by FDA.^11^ PI3K‐inhibitor umbralisib had been accelerated and approved by the FDA for refractory lymphoma on February 5, 2021, however, was withdrawn from the market on June 1, 2022, because its possible increased risk of death outweighed its benefits.^12^

Cutaneous adverse event (AE) is one of the most frequent toxicities induced by PI3K inhibitors, among which severe cutaneous AEs can weaken the quality of life (QoL) and even compromise the efficacy of treatment due to dose reductions or permanent discontinuation.^13^ Duvelisib^14^ has a black box warning for fatal or severe cutaneous AEs, while alpelisib, idelalisib, and copanlisib^15^, ^16^, ^17^ have warnings and precautions in labeling to highlight the serious risks. When administered as monotherapy, these PI3K inhibitors are associated with grade ≥ 3 rashes in up to 9% of patients.^8^ Thus, knowledge of these cutaneous AEs is indispensable for patients treated with PI3K inhibitors.

Since the overall incidence and risk of cutaneous AEs following treatment with different PI3K inhibitors have not been systemically investigated, we conducted this study to provide evidence for clinical practice.

## 2 METHODS

We followed the Preferred Reporting Items for Systematic Reviews and Meta‐Analysis (PRISMA) guidelines and prospectively registered our protocol on PROSPERO (Registration number: CRD42022320552).

### 2.1 Search strategy

We did a systematic search through ClinicalTrials.gov, PubMed, Embase, Cochrane Central Register of Controlled Trials (CENTRAL), Wanfang, China National Knowledge Infrastructure (CNKI), and China Biology Medicine disc (CBM disc) from inception to July 29, 2022. The search was conducted by combining the keywords “PI3K,” “phosphatidylinositol 3 kinase,” “alpelisib,” “idelalisib,” “copanlisib,” “duvelisib,” and “randomized controlled trial.”

### 2.2 Study selection

The inclusion criteria were as follows:

1. Phase II or phase III randomized controlled trials (RCTs);
2. RCTs with patients in the treatment arm were treated with PI3K inhibitor (single agent or in combination), and patients in the control arm were treated with non‐PI3K inhibitors (placebo or other drugs);
3. RCTs reported outcomes of cutaneous AEs.

The exclusion criteria were as follows:

1. RCTs with PI3K inhibitors in both treatment and control arms;
2. Single‐arm studies;
3. Case reports, reviews, case–control studies, and cohort studies.

### 2.3 Data extraction and quality assessment

Two reviewers independently screened titles and abstracts, examined the eligible studies, and assessed the quality of included trials. Any discrepancy was resolved by discussion or by consulting a third author. The data collected for each study included the first author, year of publication, trial design, NCT number, type of cancers, number of enrolled participants, number of available patients for safety analysis, the median age of treatment group, type of PI3K inhibitors, settings of control arms, number of all‐grade, high‐grade (grade ≥ 3), and severe cutaneous AEs in each arm. In the event of duplicates or publications reporting on the same study population, only the most recent, relevant, and the most detailed data were retained. The quality of eligible RCTs was evaluated in accordance with the Cochrane Collaboration's Risk of Bias assessment tool.^18^

### 2.4 Outcomes

The incidence and risk of all‐grade, high‐grade rash, and rash diagnosed as serious adverse events (SAEs) in patients who applied PI3K inhibitors compared with controls were primary outcomes. SAEs were defined as AEs that result in death, extended hospitalization, ongoing or significant incapacity, birth defect, or require medical or surgical intervention.^19^ The incidence and risk of all‐grade pruritus and dry skin reported in the included trials were secondary outcomes.

### 2.5 Statistical analysis

The meta‐analysis was conducted with a Review Manager (Revman, version 5.4). The pooled measures were incidence, risk ratios (RRs), and 95% confidence intervals (CIs). I^2^ statistic and Chi‐squared (χ^2^) test were used to test statistical heterogeneity among studies. RRs were pooled using random effect (I^2^ ≥ 50 or χ^2^ *p* ≤ 0.1) or fixed effect (I^2^ < 50 or χ^2^ *p* > 0.1) models. Subgroup analyses were conducted according to different PI3K‐inhibitor types, cancer types, and PI3K‐inhibitor assignations in order to explore the source of heterogeneity. For the primary outcome, a leave‐one‐out sensitivity analysis was performed to evaluate the impact of each study on the pooled outcomes. Eventually, a funnel plot was used to indicate possible publication biases.

## 3 RESULTS

### 3.1 Search results

**FIGURE 1:**
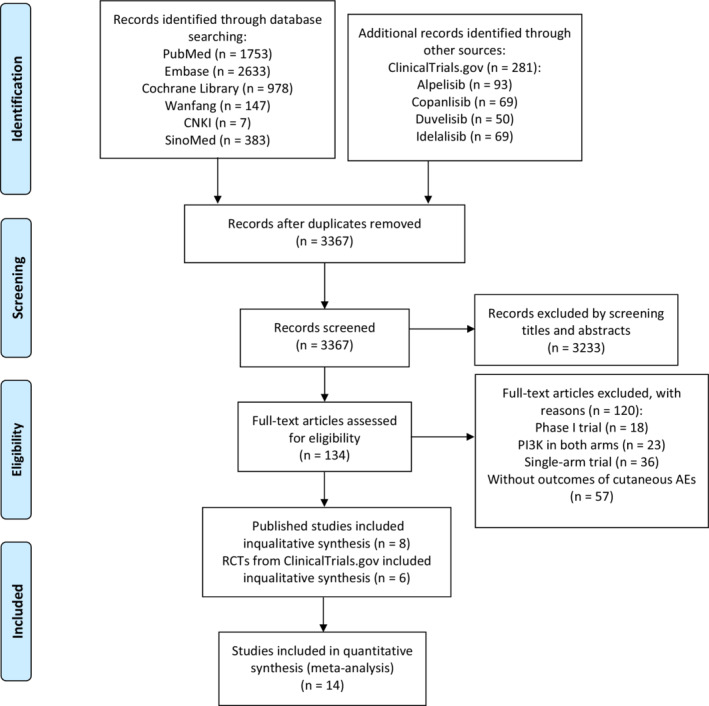
Flow diagram of studies selection

Our search identified a total of 6182 records. After preliminary screening and full‐text assessment, eight from publications^20^, ^21^, ^22^, ^23^, ^24^, ^25^, ^26^, ^27^ and six from ClinicalTrials.gov,^28^, ^29^, ^30^, ^31^, ^32^, ^33^ including 3877 participants, were retained for analyses (Figure 1).

As described in Tables 1, 2 phase II trials and 12 phase III trials were eligible for our analysis. Of the 14 RCTs, eight were on idelalisib, three were on alpelisib, two were on duvelisib, and one was on copanlisib. Seven studies were performed in patients with CCL, three in indolent non‐Hodgkin's lymphomas (iNHL), two in breast cancer (BC), one in FL, and one in head and neck cancer (HNC). Quality assessment for each study was presented in Figures S1 and S2. Nine studies used blinding methods and five were open‐label randomized trials. Overall, included trials exhibited a low risk of bias.

**TABLE 1:** Included randomized controlled trials in this study

Author/Year	ClinicalTrials.gov identifier	Phase, Trial design	Type of cancer	Type of PI3K	Enrollment	Age (treatment)	Patient groups		No. of patients for safety analysis	
							Treatment (*n*)	Control (*n*)	Treatment (*n*)	Control (*n*)
Mayer/2019	NCT01923168	2, double‐blind	BC	Alpelisib	340	64.3	Alpelisib 300 mg qd + Letrozole 2.5 mg (131)	Placebo + Letrozole 2.5 mg (126)	130	125
André/2019	NCT02437318	3, double‐blind	BC	Alpelisib	572	62.6	Alpelisib 300 mg qd + Fulvestrant (284)	Placebo + Fulvestrant (288)	284	287
Matasar/2021	NCT02367040	3, double‐blind	iNHL	Copanlisib	458	62.0	Copanlisib 60 mg + Rituximab (307)	Placebo + Rituximab (151)	307	146
Flinn/2018	NCT02004522	3, open‐label	CLL	Duvelisib	319	NA	Duvelisib (160)	Ofatumumab (159)	158	155
Sharman/2019	NCT01539512	3, double‐blind	CLL	Idelalisib	220	71	Idelalisib 150 mg bid + Rituximab (110)	Placebo + Rituximab (110)	110	108
Zelenetz/2017	NCT01569295	3, double‐blind	CLL	Idelalisib	416	62	Idelalisib 150 mg bid+ Bendamustine + Rituximab (207)	Placebo + Bendamustine + Rituximab (209)	207	209
Jones/2017	NCT01659021	3, open‐label	CLL	Idelalisib	261	67	Idelalisib 150 mg bid + Ofatumumab (174)	Ofatumumab (87)	173	86
Ghia/2020	NCT02970318	3, open‐label	CLL	Idelalisib	311	NA	Idelalisib 150 mg bid + Rituximab (118)	Bendamustine + Rituximab (35)	118	35
2018	NCT01980888	3, double‐blind	CLL	Idelalisib	311	64	Idelalisib 150 mg bid + Bendamustine + Rituximab (157)	Placebo + Bendamustine + Rituximab (154)	156	154
2018	NCT01980875	3, open‐label	CLL	Idelalisib	57	72.2	Idelalisib 150 mg bid + Ofatumumab (25)	Chlorambucil + Ofatumumab (24)	24	23
2018	NCT01732926	3, double‐blind	iNHL	Idelalisib	475	62	Idelalisib 150 mg bid + Bendamustine + Rituximab (320)	Placebo + Bendamustine + Rituximab (155)	317	155
2018	NCT01732913	3, double‐blind	iNHL	Idelalisib	295	64	Idelalisib 150 mg bid + Rituximab (198)	Placebo + Rituximab (97)	198	95
2021	NCT02204982	3, double‐blind	FL	Duvelisib	13	NA	Duvelisib 25 mg bid + Rituximab (6)	Placebo + Rituximab (7)	6	7
2020	NCT01602315	2, open‐label	HNC	Alpelisib	179	57.2	Alpelisib 300 mg qd + Cetuximab (71)	Cetuximab (35)	69	35

Abbreviations: BC, breast cancer; bid, twice daily; CCL, chronic lymphocytic leukemia; FL, follicular lymphoma; iNHL, indolent non‐Hodgkin's lymphomas; HNC, head and neck cancer; NA, not available; qd, once daily.

**TABLE 2:** Subgroup analyses for the incidence and risk of all‐grade rash related to PI3K inhibitors

Subgroup	All‐grade rash					
	No. of studies	Treatment (%)	Control (%)	RR, 95%CI, *p* value	*I* ^2^ (%), χ^2^ *p* value	χ^2^ *p* value for subgroup differences
	14	592/2257 (26.2)	176/1620 (10.9)	2.29 [1.58, 3.31], **<0.0001**	78, **<0.00001**	
PI3K inhibitor type						0.06
Alpelisib	3	185/483 (38.3)	41/447 (9.2)	3.15 [0.88, 11.31], 0.08	94, **<0.00001**	
Idelalisib	8	355/1303 (27.2)	107/865 (12.4)	2.29 [1.87, 2.81], **<0.00001**	40, 0.11	
Duvelisib	2	17/164 (10.4)	18/162 (11.1)	0.94 [0.51, 1.73], 0.83	0, 0.39	
Copanlisib	1	35/307 (11.4)	10/146 (6.8)	1.66 [0.85, 3.27], 0.14	NA	
Cancer type						**<0.00001**
iNHL	3	200/822 (24.3)	38/396 (9.6)	2.26 [1.27, 4.02], **0.005**	66, **0.05**	
BC	2	159/414 (38.4)	27/412 (6.6)	5.84 [3.98, 8.58], **<0.00001**	0, 0.86	
CCL	7	206/946 (21.8)	97/770 (12.6)	1.83 [1.46, 2.28], **<0.00001**	43, 0.11	
FL	1	1/6 (16.7)	0/7 (0)	3.43 [0.16, 71.36], 0.43	NA	
HNC	1	26/69 (37.7)	14/35 (40)	0.94 [0.57, 1.56], 0.82	NA	
PI3K inhibitor assignation						**<0.00001**
Combined anti‐CD20 antibodies^a^ with or without chemotherapy	10	391/1616 (24.2)	117/1018 (11.5)	2.24 [1.84, 2.71], **<0.00001**	26, 0.20	
Combined endocrine therapy^b^	2	159/414 (38.4)	27/412 (6.6)	5.84 [3.98, 8.58], **<0.00001**	0, 0.86	
Monotherapy	1	16/158 (10.1)	18/155 (11.6)	0.87 [0.46, 1.65], 0.82	NA	
Combined EGFR^c^	1	26/69 (37.7)	14/35 (40)	0.94 [0.57, 1.56], 0.67	NA	

*Note*: Significant differences (*p* value <0.05) between the PI3K inhibitor group and the control group are shown in **bold**.

Abbreviations: BC, breast cancer; CCL, chronic lymphocytic leukemia; CI, confidence interval; EGFR, epidermal growth factor receptor; FL, follicular lymphoma; HNC, head and neck cancer; iNHL, indolent non‐Hodgkin's lymphomas; NA, not available; PI3K, phosphatidylinositol‐3‐kinase; RR, risk ratio.

^a^ Including rituximab and ofatumumab.

^b^ Including letrozole and fulvestrant.

^c^ Including cetuximab.

### 3.2 Incidence and risk of all‐grade and high‐grade (grade ≥3) rash

**FIGURE 2:**
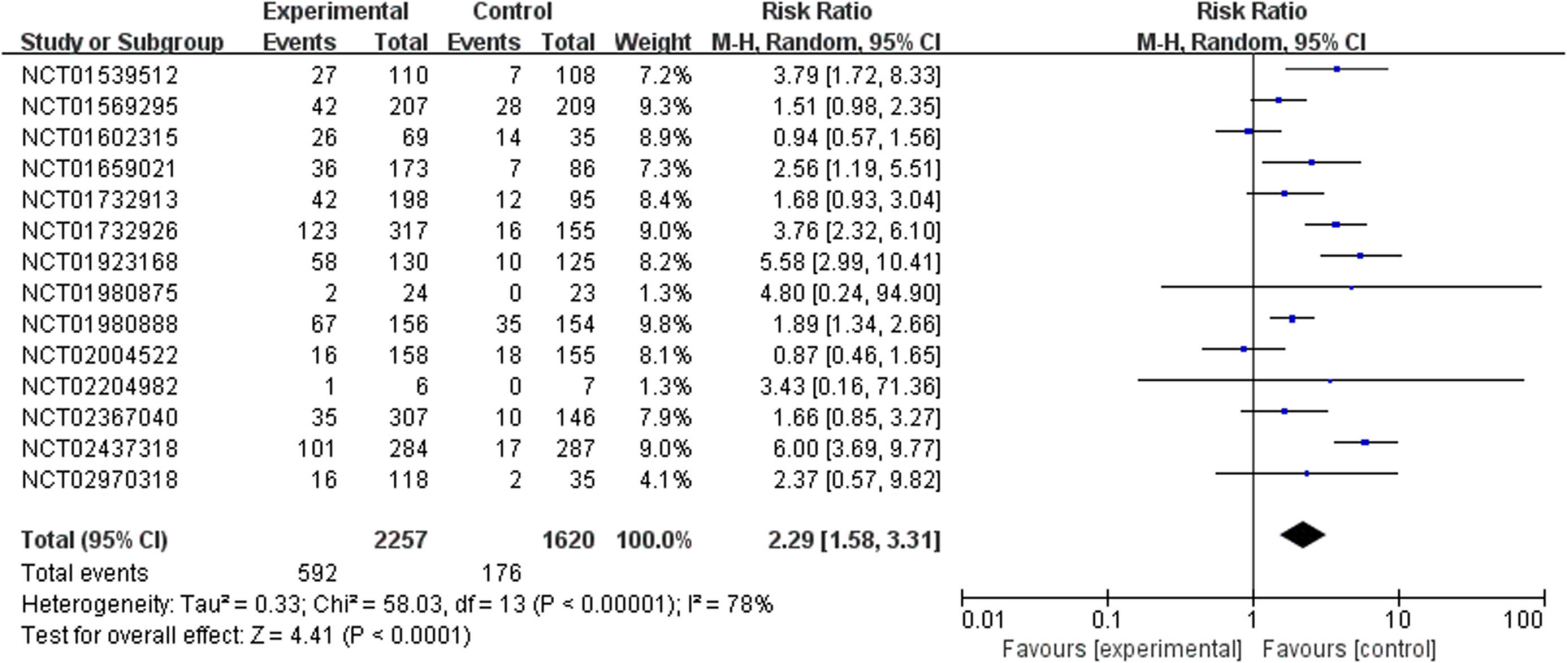
Forest plots of the risk of all‐grade rash

**FIGURE 3:**
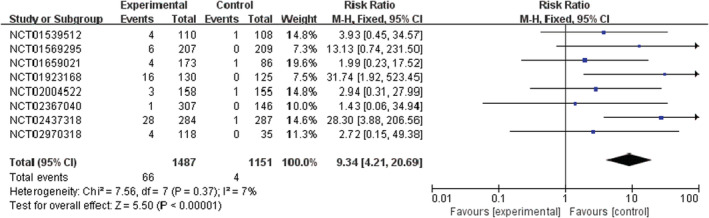
Forest plots of the risk of high‐grade rash

**FIGURE 4:**
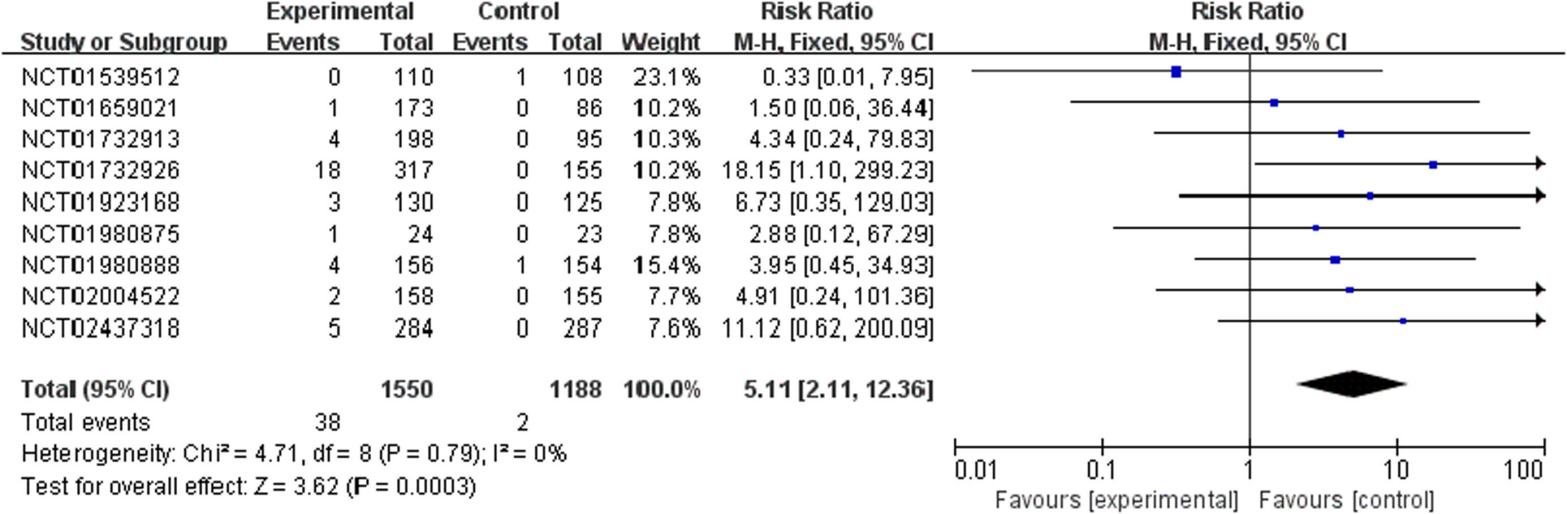
Forest plots of the risk of serious rash events

PI3K inhibitors significantly increased the risk of all‐grade and high‐grade rash compared with the control group (RR 2.29, 95% CI 1.58–3.31, *p* < 0.00001, Figure 2; RR 9.34, 95% CI 4.21–20.69, *p* < 0.00001, Figure 3). In addition, the risk of serious rash events was also observed to increase (RR 5.11, 95% CI 2.11–12.36, *p* = 0.0003, Figure 4).

In the overall population treated with PI3K inhibitors, the incidences were 26.2% (592/2257) and 4.4% (66/1487) for all‐grade and high‐grade rash, respectively. About 6.4% (38/592) of PI3K inhibitor‐related rash events were SAEs. By contrast, the incidences of all‐grade and high‐grade rash were lower in patients with the control strategy, at 10.9% (176/1620) and 0.3% (4/1151), respectively (Table S1).

### 3.3 Incidence and risk of all‐grade pruritus and dry skin

In comparison with the control group, PI3K inhibitors significantly increased the risk of pruritus and dry skin (RR 1.63, 95% CI 1.14–2.33, *p* = 0.007, Figure S3; RR 3.34, 95% CI 2.30–4.85, *p* < 0.00001, Figure S4). The incidences of pruritus and dry skin were 13.4% (284/2115) and 9.8% (141/1436) in the treatment group and 8.1% (127/1562) and 3.1% (31/988) in the control group (Table S1).

### 3.4 Other cutaneous AEs

The findings for other cutaneous AEs related to PI3K inhibitors were insufficient for a meta‐analysis, which includes alopecia (20.3%, 84/414), Stevens‐Johnson syndrome (SJS) (0.50%, 4/808), urticaria (2.52%, 30/1191), skin ulcer (1.04%, 5/480), toxic skin eruption (1.05%, 5/475), toxic epidermal necrolysis (TEN) (0.32%, 1/317), and drug reaction with eosinophilia and systemic symptoms (DRESS) (0.48%, 1/207).

### 3.5 Subgroup analyses

Significant heterogeneities were detected among the studies of all‐grade rash (*p* < 0.00001, *I* ^2^ = 78%). Therefore, subgroup analyses were performed according to different PI3K inhibitor types, cancer types, and PI3K inhibitor assignations. The results are shown in Table 2.

#### 3.5.1 Influence of different PI3K inhibitor types

Stratified by different PI3K inhibitor types, the incidences of all‐grade rash in patients with alpelisib, idelalisib, duvelisib, and copanlisib were 38.3% (185/483), 27.2% (355/1303), 10.4% (17/164), and 11.4% (35/307), respectively. Compared with the control group, idelalisib significantly increased the risk of rash (RR 2.29, 95% CI 1.87–2.81, *p* < 0.00001), whereas alpelisib increased the risk without reaching statistical significance (RR 3.15, 95% CI 0.88–11.31, *p* = 0.08). There was no statistically significant subgroup effect (*p* = 0.06).

#### 3.5.2 Influence of different cancer types

Only one trial with a small sample size was included for the FL and NHSCC each. Rash incidence was the highest in BC with observed event rates at 38.4% (159/414). Patients with CCL appeared to suffer the lowest incidence of rash at 21.8% (206/946). The increased risk of all‐grade rash were observed among trials of iNHL, BC, and CCL (RR 2.26, 95% CI 1.27–4.02, *p* = 0.005; RR 5.84, 95% CI 3.98–8.58, *p* < 0.00001; and RR 1.83, 95% CI 1.46–2.28, *p* < 0.00001). Heterogeneity existed between subgroups (*p* < 0.00001).

#### 3.5.3 Influence of different PI3K inhibitor assignations

When analyzed according to PI3K inhibitor assignations, the meta‐analysis showed that PI3K inhibitors combined with anti‐CD20 antibodies or endocrine therapy were associated with increased risks of rash (RR 2.24, 95% CI 1.84–2.71, *p* < 0.00001; RR 5.84, 95% CI 3.98–8.58, *p* < 0.00001). Only one trial each was included in a subgroup of PI3K inhibitors monotherapy and PI3K inhibitors combined EGFR, with the incidence of 10.1% and 37.7%. Heterogeneity in between‐subgroup was detected (*p* < 0.00001).

### 3.6 Sensitivity analyses and publication bias

The exclusion of every single study revealed no substantial alterations in the pooled risk of the primary outcome (Table S2). There was acceptable asymmetry in the funnel plot (RR of all‐grade rash) indicating a low risk of publication bias (Figure S5).

## 4 DISCUSSION

In this study, we assessed the overall incidence and risk of cutaneous AEs following PI3K inhibitors in cancer patients.

PI3K pathway plays a major role in several tumors, making it an attractive therapeutic target in oncology.^1^, ^2^, ^9^, ^34^, ^35^, ^36^, ^37^, ^38^ Since the first approval of idelalisib in 2014,^17^ PI3K inhibitors have provided another alternative option for cancer patients. Nowadays, four PI3K inhibitors have been approved by US FDA for clinical treatment of hematological and breast cancers.

Although these approvals have validated that these PI3K inhibitors are clinically effective, their application is limited due to some adverse effects. Cutaneous AEs appeared to be the most prevalent AEs with PI3K inhibitors. The most commonly reported cutaneous AEs related to PI3K inhibitors are rash, pruritus, and dry skin. Fatal cutaneous AEs, such as SJS, TEN, and DRESS have also been described in some trials.^25^, ^27^

Our results demonstrated a significantly increased risk of all‐grade rash, pruritus, and dry skin related to PI3K inhibitors. High‐grade rash and serious rash events were 1.22 and 1.42 times more likely to occur in patients receiving PI3K inhibitors compared to control groups, which further supported that PI3K inhibitor‐related rash might lead to deleterious effects. The incidence of high‐grade rash with alpelisib was reported as 2.8%,^39^ while it was 0.6%–2.8% with copanlisib,^40^, ^41^ 4% with idelalisib,^42^ and 7% with duvelisib^43^ according to the previous studies, which was in line with our results.

We further explored heterogeneities in rash risk stratified by types of PI3K inhibitors. There was no significant interaction detected between different PI3K inhibitors for the risk of rash. However, the *p* value in the between‐subgroup (χ^2^ *p* = 0.06) was on a borderline level of statistical significance, which indicated that PI3K inhibitor types may be associated with a rash. Our study revealed a high incidence of rash in patients receiving alpelisib or idelalisib, which was consistent with previous studies, in which rash occurred in 38%–53% and 10%–22% of patients, respectively.^39^, ^44^, ^45^ Among subjects taking alpelisib, a nonsignificant increased risk of rash events was observed, whereas a statistically increased risk was seen for patients taking idelalisib. In terms of patients taking copanlisib or duvelisib, the incidence of rash was relatively low. There are several class isoforms of PI3K inhibitors, differing from their diverse chemical structures function and isoform selectivities, which might contribute to different safety profiles of skin.^46^ PI3Kδ and PI3Kγ isoforms have been proved to play parts in T cells activation and immune response,^47^, ^48^, ^49^, ^50^, ^51^, ^52^ and targeting of these isoforms leads to activation of immune recognition.^53^, ^54^ Thus, the PI3Kδ inhibitor idelalisib was thought to be associated with immune‐mediated toxicities in the lymphocytes of the epidermis or dermis, such as a rash.^55^ While PI3Kα was considered to express lowly in immune cells,^56^ patients with rash receiving PI3Kα inhibitor alpelisib had a trend toward increased blood eosinophils,^57^ which might support the occurance of dermal hypersensitivity reaction. On the other hand, the nonsignificant results of PI3K⍺/δ inhibitor copanlisib and PI3Kγ/δ inhibitor duvelisib may be explained by the limited number and small sample size of included trials. Thus, the potential risks should not be ignored and further studies based on large random clinical trials are needed.

Due to the limited efficacy as single agents, the PI3K inhibitors are usually applied in combination with other drugs. We also evaluated the risk of rash for several subgroups of different PI3K inhibitor assignations and found that PI3K inhibitor assignations were important variables (χ^2^ *p* < 0.00001). A significant increase in the risk of rash related to PI3K inhibitors combined endocrine therapy or anti‐CD20 antibodies was observed. Furthermore, our pooled analysis of rash incidence also demonstrated that the rates were higher in patients receiving PI3K inhibitor combination regimens. Conversely, it is underpowered to identify whether the nonsignificant rash risk is attributed to duvelisib monotherapy or is merely the result of limited sample size. There is accumulating evidence indicating that anti‐CD20 antibodies used for tumor can lead to cutaneous AEs such as rash, with reported rates at 10%–17%.^58^, ^59^, ^60^, ^61^ The original drug signaling pathway and the PI3K pathway were considered to have potential interaction, for as part of combination regimens, the PI3K inhibitor had the power to enhance sensitivity of other treatments,^62^, ^63^, ^64^ which may explain the increased risk of accompanying cutaneous AEs.

Notably, the time to onset (TTO) of PI3K inhibitor‐related cutaneous AEs was ranged from 1 to 2 months from according to some case reports,^65^, ^66^ while a cohort study suggested that the median TTO of alpelisib‐associated rash was 2 weeks.^67^

Given the widespread use of PI3K inhibitors and the negative impact of cutaneous AEs on both QoL and therapeutic effectiveness, it is important to recognize the potential risk of these cutaneous AEs associated with PI3K inhibitors. The recommended management for mild cutaneous AEs includes the use of topical corticosteroids, antihistamines, antibiotics, antipruritic agents, fragrance‐free soaps, and mild moisturizers.^51^

The mechanisms of PI3K inhibitor‐associated cutaneous AEs are still unclear. Histologic manifestations of PI3K inhibitor‐related rash are featured with skin hypersensitivity reactions,^47^, ^55^, ^68^ which means that appearance of cutaneous AEs are direct results of PI3K inhibition and subsequent enhanced immune system activation. It was further proposed that inhibition of PI3K signaling might result in structural changes to the epidermis and dermis, leading to epidermal cell death^57^, ^69^ and performing as related cutaneous AEs. These speculations might explain the mechanisms of PI3K inhibitor‐related cutaneous AEs.

Our study presents several limitations. First, the included studies were performed at various medical centers, thus the potential bias in detection rates of cutaneous AEs cannot be ignored. Second, high heterogeneity was detected in all‐grade rash and pruritus, which may indicate that the results obtained were not reliable enough. However, we performed subgroup analyses and found that the different PI3K‐inhibitor assignations, cancer types, and differences in sample size might be the possible sources of heterogeneity. Third, six RCTs from ClinicalTrials.gov were not classified by Common Terminology Criteria for Adverse Events (CTCAE), leaving missing data of high‐grade events. Fourth, the included RCTs were not specifically conducted for the assessment of AEs, leading to an underestimation of PI3K inhibitor‐related cutaneous AEs. Furthermore, some publications only reported AEs above a certain threshold (e.g., >5% or 10%), which could lead to the misestimation of the estimated incidences of cutaneous AEs. However, we searched and extracted cutaneous AEs from ClinicalTrials.gov to minimize publication bias to the greatest extent. Moreover, some data were abstracted from ClinicalTrials.gov, so we did not have access to the duration of follow‐up of the study population, which may be an important factor influencing the incidence of cutaneous AEs. Last, due to lacking evidence of existing studies, it is difficult to make comprehensive and accurate conclusions about the risk factors and clinical characteristics of PI3K inhibitor‐associated cutaneous AEs. Hence, studies focused on safety profiles of PI3K inhibitor‐associated cutaneous AEs are further needed.

## 5 CONCLUSION

Our meta‐analysis demonstrated that cancer patients using PI3K inhibitors were associated with a significant higher risk of rash, pruritus, and dry skin compared with controls. The increased risk of high‐grade rash and severe rash events were also detected. Prompt identification and effective management is necessary because high‐grade and severe cutaneous AEs can be life‐threatening. Future research are required to investigate factors and pathogenesis that potentially contribute to PI3K inhibitor‐associated cutaneous AEs in the population of cancer patients.

## AUTHORS’ CONTRIBUTION

X.J. Yu and X. Feng were arbiters of disagreement and responsible for the conceptualization and supervision of the study. Y.S. Wang, Z. Ma, and Y. Zhang did the data acquisition and interpretation. Y.S. Wang drafted the writing of the manuscript with revisions based on Z. Ma and Z.L. An's comments. All authors did a critical review of the final version.

## FUNDING INFORMATION

None.

## CONFLICT OF INTEREST

All authors declare that they have no conflict of interest.

## Supporting information


Appendix S1

## Data Availability

All data are available within the manuscript and supplemental materials.
