# Supplementary material for: Risk of cutaneous adverse events in cancer patients treated with phosphatidylinositol‐3‐kinase inhibitors: A systematic review and meta‐analysis of randomized controlled trials
Source: Cancer Med. 2022 Aug 19;12(3):2227–37. doi: 10.1002/cam4.5153 (PMC9939201; doi:10.1002/cam4.5153)
Supplement: Supplementary file 1 — Appendix S1 [file CAM4-12-2227-s001.docx]

**SUPPLEMENTARY MATERIAL**

**Supplementary Table 1** The cases of cutaneous AEs extracted from included studies

| **ClinicalTrials.gov Identifier** | **All-Grade Rash^*^** | | **High-Grade (Grade ≥ 3) Rash^*^** | | **Serious Rash^*^ Event** | | **All-Grade Pruritus** | | **All-Grade Dry Skin** | |
| --- | --- | --- | --- | --- | --- | --- | --- | --- | --- | --- |
|  | **Intervention** | **Control** | **Intervention** | **Control** | **Intervention** | **Control** | **Intervention** | **Control** | **Intervention** | **Control** |
| NCT01923168 | 58 | 10 | 16 | 0 | 3 | 0 | 24 | 9 | 19 | 4 |
| NCT02437318 | 101 | 17 | 28 | 1 | 5 | 0 | 61 | 16 | 42 | 10 |
| NCT02367040 | 35 | 10 | 1 | 0 | 0 | 0 | 30 | 9 | 17 | 1 |
| NCT02004522 | 16 | 18 | 3 | 1 | 2 | 0 | 11 | 9 | 0 | 0 |
| NCT01539512 | 27 | 7 | 4 | 1 | 0 | 1 | 6 | 5 | 0 | 0 |
| NCT01569295 | 42 | 28 | 6 | 0 | 0 | 0 | 17 | 12 | 0 | 0 |
| NCT01659021 | 36 | 7 | 4 | 1 | 1 | 0 | 21 | 6 | 11 | 1 |
| NCT02970318 | 16 | 2 | 4 | 0 | 0 | 0 | 0 | 0 | 0 | 0 |
| NCT01980888 | 67 | 35 | / | / | 4 | 1 | 24 | 32 | 14 | 4 |
| NCT01980875 | 2 | 0 | / | / | 1 | 0 | 0 | 0 | 0 | 0 |
| NCT01732926 | 123 | 16 | / | / | 18 | 0 | 54 | 22 | 20 | 5 |
| NCT01732913 | 42 | 12 | / | / | 4 | 0 | 25 | 4 | 0 | 0 |
| NCT02204982 | 1 | 0 | / | / | 0 | 0 | 0 | 1 | 0 | 0 |
| NCT01602315 | 26 | 14 | / | / | 0 | 0 | 11 | 2 | 18 | 6 |
| Risk ratio (95%CI) | 2.29 [1.58, 3.31] | | 9.34 [4.21, 20.69] | | 5.11 [2.11, 12.36] | | 1.63 [1.14, 2.33] | | 3.34 [2.30, 4.85] | |

Abbreviation:^*^included the preferred terms: dermatitis, dermatitis acneiform, dermatitis psoriasiform, drug eruption, eyelid rash, genital rash, mucocutaneous rash, perineal rash, rash, rash erythematous, rash follicular, rash generalized, rash macular, rash maculo-papular, rash maculovesicular, rash morbilliform, rash nodular, rash papular, rash papulosquamous, rash pruritic, rash pustular, rash vesicular, and rash vulvovaginal; CI, confidence interval; /, not available

**Supplementary Table 2** Sensitive analyses of included studies

| Excluded study | Risk ratio | 95% CI | I^2^, χ^2^ P value | P-value |
| --- | --- | --- | --- | --- |
| / | 2.29 | 1.58, 3.31 | 78%, < 0.00001 | P < 0.00001 |
| NCT01923168 | 2.11 | 1.46, 3.04 | 75%, < 0.00001 | P < 0.0001 |
| NCT02437318 | 2.06 | 1.47, 2.88 | 69%, 0.0001 | P < 0.0001 |
| NCT02367040 | 2.36 | 1.59, 3.50 | 79%, < 0.00001 | P < 0.0001 |
| NCT02004522 | 2.49 | 1.72, 3.59 | 76%, < 0.00001 | P < 0.00001 |
| NCT01539512 | 2.20 | 1.50, 3.24 | 79%, < 0.00001 | P < 0.0001 |
| NCT01569295 | 2.39 | 1.60, 3.57 | 78%,< 0.00001 | P < 0.0001 |
| NCT01659021 | 2.27 | 1.53, 3.37 | 79%, < 0.00001 | P < 0.0001 |
| NCT02970318 | 2.29 | 1.56, 3.35 | 79%, < 0.00001 | P < 0.0001 |
| NCT01980888 | 2.34 | 1.54, 3.58 | 79%, < 0.00001 | P < 0.0001 |
| NCT01980875 | 2.27 | 1.56, 3.29 | 79%, < 0.00001 | P < 0.0001 |
| NCT01732926 | 2.18 | 1.48, 3.22 | 77%, < 0.00001 | P < 0.0001 |
| NCT01732913 | 2.36 | 1.58, 3.51 | 79%, < 0.00001 | P < 0.0001 |
| NCT02204982 | 2.28 | 1.57, 3.31 | 79%, < 0.00001 | P < 0.0001 |
| NCT01602315 | 2.49 | 1.74, 3.57 | 73%, < 0.0001 | P < 0.00001 |

Abbreviation: CI, confidence interval.

**Supplementary Figure 1-2** Risk of bias assessment of included studies

(A) Risk of bias graph: review authors’ judgements about each risk of bias item presented as percentages across all included studies.

(B) Risk of bias summary: review authors’ judgements about each risk of bias item for each included study
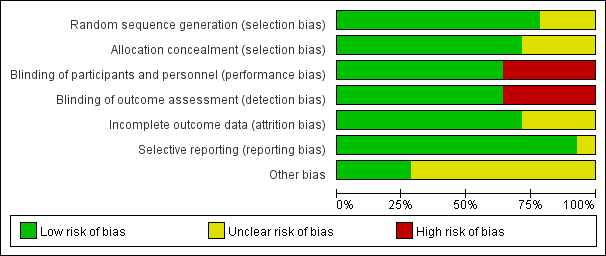

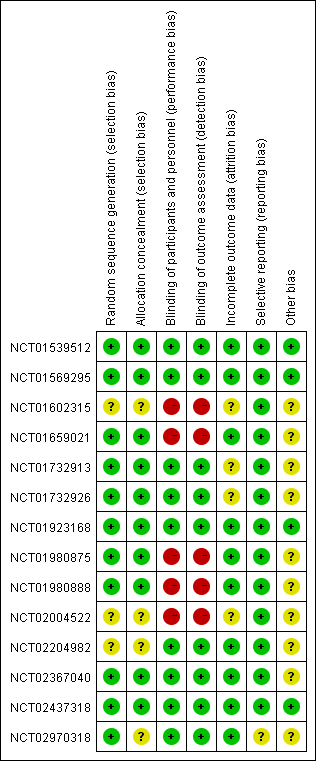


**
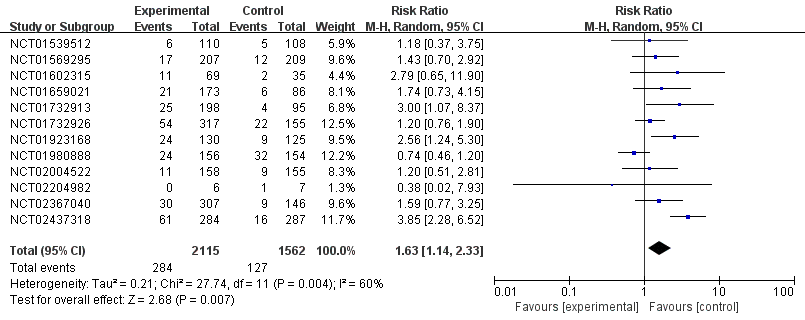
Supplementary Figure 3** Forest plots of the risk of all-grade pruritus

**Supplementary Figure 4** Forest plots of the risk of all-grade dry skin

**
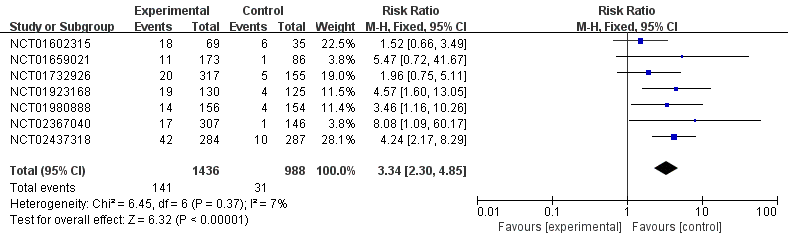
**

**Supplementary Figure 5** Funnel plots of eligible studies in the meta-analysis

**
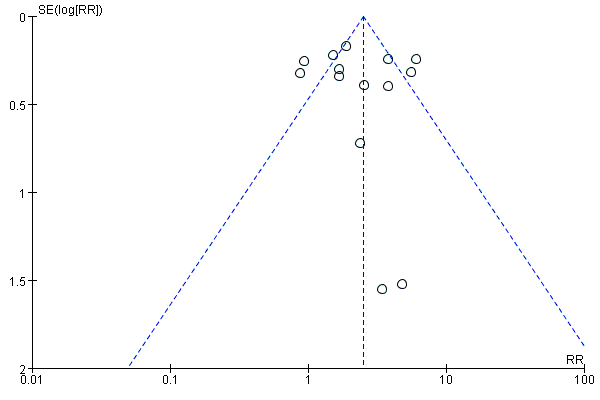
**
